# Impact of normative brain volume reports on the diagnosis of neurodegenerative dementia disorders in neuroradiology: A real-world, clinical practice study

**DOI:** 10.3389/fnagi.2022.971863

**Published:** 2022-10-12

**Authors:** Dennis M. Hedderich, Benita Schmitz-Koep, Madeleine Schuberth, Vivian Schultz, Sarah J. Schlaeger, David Schinz, Christian Rubbert, Julian Caspers, Claus Zimmer, Timo Grimmer, Igor Yakushev

**Affiliations:** ^1^Department of Neuroradiology, Klinikum rechts der Isar, School of Medicine, Technical University of Munich, Munich, Germany; ^2^Department of Diagnostic and Interventional Radiology, Medical Faculty, University Dusseldorf, Dusseldorf, Germany; ^3^Department of Psychiatry and Psychotherapy, Klinikum rechts der Isar, Sch, Munich, Germany; ^4^Department of Nuclear Medicine, Klinikum rechts der Isar, School of Medicine, Technical University of Munich, Munich, Germany

**Keywords:** Alzheimer’s disease, magnetic resonance imaging, positron-emission-tomography, biomarkers, neurodegenerative disorder (NDD), artificial intelligence—AI

## Abstract

**Background:** Normative brain volume reports (NBVR) are becoming more available in the work-up of patients with suspected dementia disorders, potentially leveraging the value of structural MRI in clinical settings. The present study aims to investigate the impact of NBVRs on the diagnosis of neurodegenerative dementia disorders in real-world clinical practice.

**Methods:** We retrospectively analyzed data of 112 memory clinic patients, who were consecutively referred for MRI and 18F-fluorodeoxyglucose (FDG) positron emission tomography (PET) during a 12-month period. Structural MRI was assessed by two residents with 2 and 3 years of neuroimaging experience. Statements and diagnostic confidence regarding the presence of a neurodegenerative disorder in general (first level) and Alzheimer’s disease (AD) pattern in particular (second level) were recorded without and with NBVR information. FDG-PET served as the reference standard.

**Results:** Overall, despite a trend towards increased accuracy, the impact of NBVRs on diagnostic accuracy was low and non-significant. We found a significant drop of sensitivity (0.75–0.58; *p* < 0.001) and increase of specificity (0.62–0.85; *p* < 0.001) for rater 1 at identifying patients with neurodegenerative dementia disorders. Diagnostic confidence increased for rater 2 (*p* < 0.001).

**Conclusions:** Overall, NBVRs had a limited impact on diagnostic accuracy in real-world clinical practice. Potentially, NBVR might increase diagnostic specificity and confidence of neuroradiology residents. To this end, a well-defined framework for integration of NBVR in the diagnostic process and improved algorithms of NBVR generation are essential.

## Introduction

Magnetic Resonance Imaging (MRI) plays a key role in the diagnostic work-up of neurodegenerative dementia disorders (Frisoni et al., 2010; Teipel et al., 2015). Besides ruling out any treatable causes for dementia (e.g., normal pressure hydrocephalus, brain tumor, etc.), the identification and characterization of regional atrophy patterns are key for guiding the diagnostic process (Teipel et al., 2015, 2017). To date, this is mostly performed visually, leading to potentially subjective results at high intra- and inter-rater variability and may depend on the radiologist’s level of expertise (Vernooij et al., 2019; Hedderich et al., 2020).

One of the most prominent use cases of Artificial Intelligence (AI) based solutions in neuroradiology has made it possible to integrate whole brain volumetry into the clinical workflow (Pemberton et al., 2021b). As we have learned more and more about brain development and aging over the lifespan from analyses of large-scale aggregated spectrum data, the reliable identification of deviations from the norm comes within reach (Bethlehem et al., 2022; Rutherford et al., 2022). This can be done by normative brain volume reports (NBVRs), which compare measured volumes of different brain structures with a healthy cohort after adjusting for sex and age, and might lead the way toward a more objective evaluation of regional brain atrophy (Potvin et al., 2017; Bruun et al., 2019). These NBVRs can present deviations from normal tissue volumes either as points plotted against a normal distribution and standard deviations or by color-coded, whole brain statistical parametric maps (SPM). A similar approach has been introduced to 18F-fluorodeoxyglucose positron emission tomography (FDG-PET) imaging of the brain more than two decades ago (Minoshima et al., 1995). Stereotactic surface projections were shown to increase diagnostic accuracy in the work-up of patients with suspected neurodegenerative disorders (Brown et al., 2014). Investigating the impact of NBVRs on the differential diagnosis of neurodegenerative disorders in a pre-selected group of patients, we found improved diagnostic accuracy and improved interrater reliability (Hedderich et al., 2020). However, investigations of CE-marked NBVRs for an unselected, chronologically defined patient cohort are still lacking.

Assessment of cerebral glucose metabolism by FDG-PET is the imaging modality of choice to rule out a neurodegenerative disorder in patients presenting with cognitive decline. However, MRI is usually performed beforehand in order to rule out treatable causes of dementia. It would be desirable to enhance the interpretation of MRI by NBVRs with respect to predicting a pathological pattern on FDG-PET. Thus, we chose FDG-PET as the reference standard, which was available by design in all included patients that were referred to our department for PET-MRI due to a suspected neurodegenerative dementia disorder. We hypothesize that, since MRI and FDG-PET share some amount of information about local brain atrophy, we can approximate the diagnostic value of MRI with NBVRs as an advanced postprocessing technique. The purpose of this study is to analyze the diagnostic accuracy of MRI compared to FDG-PET as the reference standard with respect to: (i) identifying the presence of any neurodegenerative disorder; and (ii) identifying a pattern suggestive of Alzheimer’s disease (AD) without and with NBVRs. We do so in a chronologically defined, consecutive cohort imaged at a hybrid PET-MRI system for suspected neurodegenerative dementia disorders.

## Methods

### Study cohort and design

**Figure 1:**
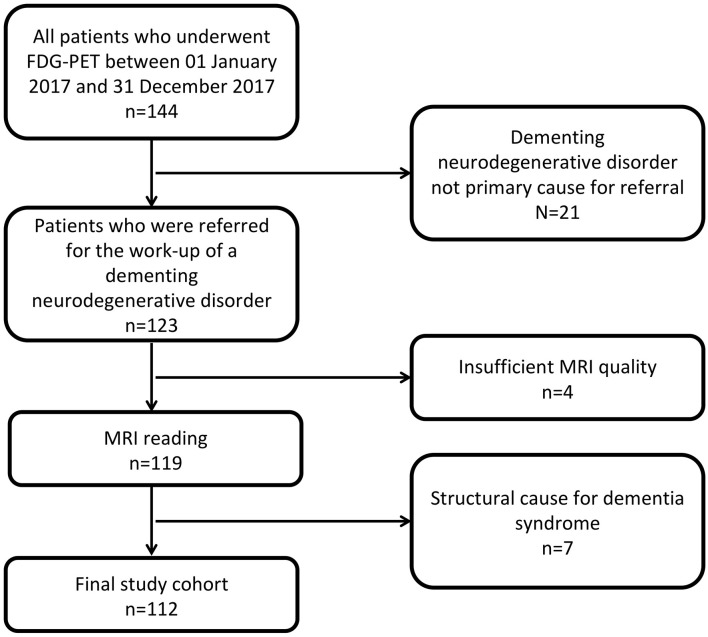
STARD patient flow diagram after application of exclusion criteria.

We retrospectively analyzed a series of consecutive patients, who were referred to the Department of Nuclear Medicine for the imaging work-up of a suspected dementia disorder between 01/01/2017 and 31/12/2017. Besides an available PET/MRI examination (see below), the inclusion criteria were: sufficient quality of structural brain MRI including a 3D-T1 gradient echo sequence with a resolution of 1 × 1 × 1 mm^3^ [e.g., Magnetization Prepared Rapid Acquisition Gradient Echo (MPRAGE)], referral for suspected neurodegenerative dementia disorder, and absence of alternative disorders causing dementia (e.g., normal pressure hydrocephalus). Detailed information about patient flow in the study can be found in Figure 1.

### Image acquisition and analysis

Imaging data were acquired on a fully integrated Siemens Biograph mMR (Siemens Medical Solutions, Knoxville, USA) PET/MR system as described in detail elsewhere (Yakushev et al., 2022). Briefly, PET data were acquired in list mode over 15 min, 30 min after an intravenous injection of approximately 185 MBq 18F-FDG. A high-resolution structural MRI sequence (T1-weighted MPRAGE) was acquired with the following parameters: TR = 2,300 ms, TE = 2.98 ms, TI = 900 ms, flip angle = 9°, acquisition matrix = 256 × 240 mm^2^, voxel size = 1 × 1 × 1 mm^3^.

NBVRs were produced using the CE-marked AI-platform BIOMETRICA (jung diagnostics GmbH, Hamburg, Germany) as described previously (Hedderich et al., 2020). T1 MRI images were segmented using a previously described and validated atlas-based volumetry approach implemented in SPM12 (Huppertz et al., 2010; Opfer et al., 2016). In brief, MRI brain scans are segmented into tissue class component images representing either gray matter (GM), white matter (WM), or cerebrospinal fluid (CSF). The total intracranial volume (TIV) is estimated using a method which was recently introduced and validated by Malone et al. (2015). Results of the tissue segmentation are visually checked for segmentation errors. All tissue segmentations passed quality control. Hereafter, standard voxel-based morphometry (VBM; Ashburner and Friston, 2000) as provided by the SPM12 software package is applied to the individual GM tissue class component image of a patient using a modification of Mühlau et al. (2009) for asymmetric statistical designs. The scanner- and sequence-specific normative database comprised 26 healthy subjects with a mean age of 57 years (standard deviation of 11 years) ranging from 41 to 81 years. Spatial correspondence between the individual GM tissue class component image of the patient and the GM tissue class component images of the normative database is established *via* a high-dimensional nonlinear image registration technique (DARTEL; Ashburner, 2007). GM volumes on voxel level are adjusted for TIV and age to minimize the impact of these confounding variables on statistical analysis. The adjustment is performed by computing the residuals from a bilinear regression function. Voxel-wise *t*-tests of age- and TIV-adjusted GM volumes between patients and healthy individuals are performed. An extent threshold of 125 voxels corresponding to a cluster volume of 1 ml is set to partially correct for multiple comparisons (Forman et al., 1995). The resulting *p*-values are presented as color-coded overlay on axial slides and surface projections.

### Image reading

FDG-PET images were read by a nuclear medicine physician (IY) with about 10 years of experience and special training in brain imaging. The ratings served as the reference standard. The rater was blind to all clinical information except for age. Axial FDG-PET images, along with 3D-SSP maps (Minoshima et al., 1995), were rated as following. First, images of each subject were rated as either indicative of a neurodegenerative dementia disorder or not. In the former case, the subject was subsequently rated as either indicative of AD or not. The AD-positive pattern included reduced FDG uptake in substantial parts of the lateral and/or mesial parietal cortex, as well as in the lateral temporal cortex, with sparing of the sensorimotor cortex, basal ganglia, and the cerebellum.

**Figure 2:**
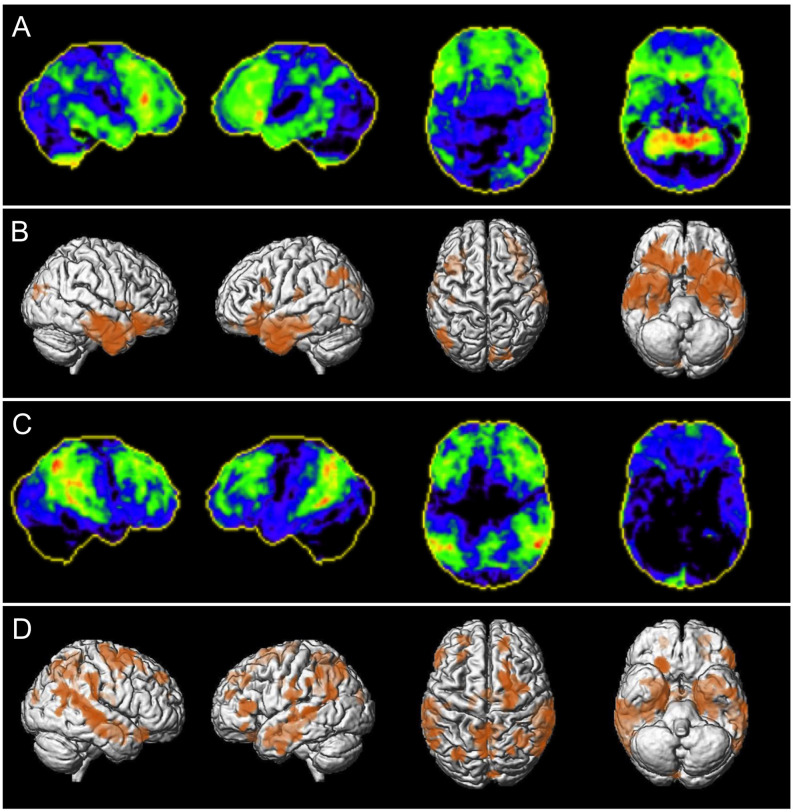
Patient examples of correctly classified neurodegenerative disease using NBVRs. Panels **(A,C)** depict negative z-score deviations of FDG-PET imaging using Neurostat (warmer colors represent larger negative z-scores). Panels **(B,D)** depict 3D renderings of age-adjusted gray matter volume deficits using voxel-based morphometry at *p* < 0.005 uncorrected. Panels **(A,B)** show a 70-year-old female patient who was initially falsely evaluated as non-neurodegeneration by both raters and then correctly classified as showing signs of neurodegenerative disease using NBVRs. Panels **(C,D)** show a 56-year-old male patient who was initially falsely evaluated as non-neurodegeneration by rater 1 and then correctly classified as showing signs of neurodegenerative disease using NBVRs. Abbreviations: NBVR, Normative brain volume report.

All brain MRI images and NBVRs were evaluated by two neuroradiologists in two sessions. Rater 1 was a neuroradiology resident and board-certified neurologist with 2 years of neuroimaging experience in evaluating patients with suspected neurodegenerative dementia disorder. Rater 2 was a neuroradiology resident with 3 years of experience. The only clinical information available to the reviewers were sex and age, ratings were performed blinded for all other clinical or biomarker information. Visual assessment of regional brain atrophy was based on axial, coronal, and sagittal reconstructions of the 3D T1-weighted MRI sequence at 1 mm^3^ isotropic resolution. The raters were able to adapt image reconstructions. Obviously, raters were not aware of the distribution of diagnoses within the study cohort. The evaluation took place in two reading sessions. All brain MRI scans were evaluated both with and without an NBVR in two reading sessions by both raters independently. The order of the two types of evaluation was assigned randomly to exclude training effects. The two reading sessions were scheduled 4 weeks apart, in order to exclude a memory bias. Raters did not receive a study-specific training to assess brain regional atrophy patterns due to their strong clinical background in neuroradiology and to resemble clinical routine. Both raters had to state: (i) whether there is abnormal brain volume loss present, suggestive of any neurodegenerative disease; (ii) whether the atrophy pattern is suggestive of AD; and (iii) how confident they were in their respective rating. For detection and differential diagnosis of brain atrophy patterns, the readers interpreted the SPMs of GM volume deviations from the normal control cohort, presented as both axial slices and 3D renderings at *p* < 0.005, uncorrected (see Figure 2 for exemplary 3D renderings). An AD-type atrophy pattern was defined as follows: symmetric or asymmetric atrophy of the medial temporal lobe, temporoparietal junction (TPJ), and posterior cingulate cortex (PCC). Frontal atrophy was facultative (Whitwell et al., 2011).

### Statistical analysis

Individual rating results were analyzed using crosstables. Sensitivity, specificity, positive predictive value, and negative predictive value including 95% confidence intervals were calculated. Differences in intra-individual correct classifications between visual inspection alone and visual evaluation plus NBVR were calculated using McNemar’s test. Differences in diagnostic confidence were evaluated using Wilcoxon’s signed rank test. Differences were considered statistically significant for *p* < 0.05. To assess interrater reliability, Cohen’s kappa was calculated. All statistical tests were performed using SPSS version 26.0 (SPSS, IBM Corp. 2017).

## Results

### Study cohort

After the application of exclusion criteria (Figure 1), imaging data of *n* = 112 patients were evaluated. Their mean age was 63.9 ± 13.8 years, 63 (56.3%) were male. FDG-PET images of 57 subjects (50.9%) were rated as indicative of a neurodegenerative dementia disorder. Among them, 31 (54.4%) were deemed suggestive of AD.

### Diagnostic accuracy for detection of neurodegenerative disease

The diagnostic accuracy for the detection of patients with neurodegenerative dementia disorders did not differ significantly between the two reading conditions for rater 1 [visual inspection only: 0.69 (0.60–0.77), visual inspection plus NBVR: 0.71 (0.63–0.80)] and rater 2 [visual inspection only: 0.66 (0.57–0.75), visual inspection plus NBVR: 0.69 (0.60–0.77)].

Conventional visual inspection of brain MRI for the identification of patients with neurodegenerative disease yielded sensitivities and specificities of 0.75 (95%-CI: 0.62–0.85)/0.62 (0.48–0.74) and 0.51 (0.37–0.64)/0.82 (0.69–0.90) for raters 1 and 2, respectively. Detection of the presence of any neurodegenerative pattern based on visual evaluation and NBVRs yielded sensitivities and specificities of 0.58 (0.41–0.60)/0.85 (0.73–0.93) and 0.56 (0.42–0.69)/0.82 (0.69–0.90) for raters 1 and 2, respectively. When comparing classification results without and with NBVRs, we found a significant drop in sensitivity (*p* < 0.001) and increase in specificity (*p* < 0.001) for rater 1, whereas no statistical difference was found for rater 2. Additional metrics of diagnostic accuracy can be found in Table 1. Crosstable analysis as well as confidence ratings of the identification of any neurodegenerative disorder are shown in Table 2. In order to illustrate our results, two examples of patients who were initially judged false negative by visual inspection alone and classified correctly as patients with neurodegenerative disease using NBVRs are shown in Figure 2.

**Table 1:** Metrics of diagnostic accuracy including 95% confidence intervals for raters 1 and 2.

**Rater**	**Question**	**Mode**	**Accuracy**	**Sensitivity**	**Specificity**	**PPV**	**NPV**
1	Neurodegeneration	Visual inspection	0.69 (0.60–0.77)	0.75 (0.62–0.85)	0.62 (0.48–0.74)	0.67 (0.54–0.78)	0.71 (0.56–0.83)
		Visual inspection + NBVR	0.71 (0.63–0.80)	0.58 (0.41–0.60)	0.85 (0.73–0.93)	0.80 (0.65–0.91)	0.67 (0.54–0.77)
	AD	Visual inspection	0.71 (0.57–0.84)	0.79 (0.57–0.92)	0.60 (0.36–0.80)	0.61 (0.46–0.75)	0.39 (0.25–0.54)
		Visual inspection + NBVR	0.77 (0.63–0.91)	0.78 (0.52–0.93)	0.76 (0.50–0.92)	0.51 (0.34–0.68)	0.49 (0.32–0.66)
2	Neurodegeneration	Visual inspection	0.66 (0.57–0.75)	0.51 (0.37–0.64)	0.82 (0.69–0.90)	0.74 (0.58–0.86)	0.62 (0.49–0.73)
		Visual inspection + NBVR	0.69 (0.60–0.77)	0.56 (0.42–0.69)	0.82 (0.69–0.90)	0.76 (0.60–0.87)	0.64 (0.52–0.75)
	AD	Visual inspection	0.63 (0.46–0.81)	0.79 (0.49–0.94)	0.50 (0.26–0.75)	0.63 (0.44–0.79)	0.37 (0.21–0.56)
		Visual inspection + NBVR	0.56 (0.39–0.73)	0.67 (0.39–0.87)	0.47 (0.25–0.71)	0.59 (0.41–0.75)	0.41 (0.25–0.59)

Abbreviations: AD, Alzheimer’s disease; NBVR, normative brain volume report; NPV, negative predictive value; PPV, positive predictive value.

**Table 2:** Identification of individuals with and without atrophy patterns suggestive of neurodegenerative disease.

			**Reference standard FDG-PET**	
			**ND present**	**ND absent**
Rater 1	Visual inspection	ND present	43 [1 (1–2)]	21 [1 (1–1.5)]
		ND absent	14 [2 (1–1.5)]	34 [1 (1–2)]
	Visual inspection + NBVR	ND present	33 [1 (1–1.5)]	8 [1.5 (1–2)]
		ND absent	24 [1 (1–2)]	47 [1 (1–2)]
Rater 2	Visual inspection	ND present	29 [1 (1–1.5)]	10 [2 (2–2)]
		ND absent	28 [2 (1–2)]	45 [1 (1–2)]
	Visual inspection + NBVR	ND present	32 [1 (1–1)]	10 [1 (1–2)]
		ND absent	25 [1 (1–2)]	45 [1 (1–1)]

Crosstable analyses of readers 1 and 2 without and with NBVRs are shown. Confidence ratings (1 = high, 2 = medium, 3 = low diagnostic confidence) are given in parentheses [median (first quartile − third quartile)]. Abbreviations: NBVR, normative brain volume report; ND, neurodegeneration.

### Diagnostic accuracy for identification of Alzheimer’s disease

The diagnostic accuracy for the detection of patients with AD did not differ significantly between the two reading conditions for rater 1 [visual inspection only: 0.71 (0.57–0.84), visual inspection plus NBVR: 0.77 (0.63–0.91)] and rater 2 [visual inspection only: 0.63 (0.46–0.81), visual inspection plus NBVR: 0.56 (0.39–0.73)].

Among the patients with neurodegenerative disorders, diagnostic accuracy for the identification of AD was evaluated. Conventional visual inspection of brain MRI yielded sensitivities and specificities of 0.79 (95%-CI: 0.57–0.92)/0.79 (0.49–0.94) and 0.60 (0.36–0.80)/0.5 (0.26–0.74) for raters 1 and 2, respectively. Detection of the AD typical patterns based on visual evaluation and NBVRs yielded sensitivities and specificities of 0.78 (0.52–0.93)/0.76 (0.50–0.92) and 0.67 (0.39–0.87)/0.47 (0.25–0.71) for raters 1 and 2, respectively. No statistically significant differences between visual ratings and NBVR-supported ratings were found. For additional metrics of differential diagnostic accuracy, please see Table 1. Crosstable analysis, as well as confidence ratings of the identification of an FDG-PET pattern suggestive of AD, are shown in Table 3.

**Table 3:** Differential diagnosis of between individuals with a pattern of AD and non-AD neurodegeneration.

			**Reference standard FDG-PET**	
			**AD pattern**	**Non-AD pattern**
Rater 1	Visual inspection	AD pattern	19 [1 (1–2)]	8 [1.5 (1–2)]
		Non-AD pattern	5 [1 (1–2)]	12 [1 (1–2)]
	Visual inspection + NBVR	AD pattern	14 [2 (1–2)]	4 [1 (1–1.75)]
		Non-AD pattern	4 [1.5 (1–2)]	13 [1 (1–2)]
Rater 2	Visual inspection	AD pattern	11 [2 (1–2)]	8 [2 (1.25–2.75)]
		Non-AD pattern	3 [2 (2–2)]	8 [1 (1–2)]
	Visual inspection + NBVR	AD pattern	10 [2 (1.75–2)]	10 [2 (1–2.25)]
		Non-AD pattern	5 [2 (2–2)]	9 [2 (1–2.5)]

Crosstable analyses of readers 1 and 2 without and with NBVRs are shown. Confidence ratings (1 = high, 2 = medium, 3 = low diagnostic confidence) are given in parentheses [median (first quartile − third quartile)]. Abbreviations: AD, Alzheimer’s disease; NBVR, normative brain volume report.

### Diagnostic confidence

**Figure 3:**
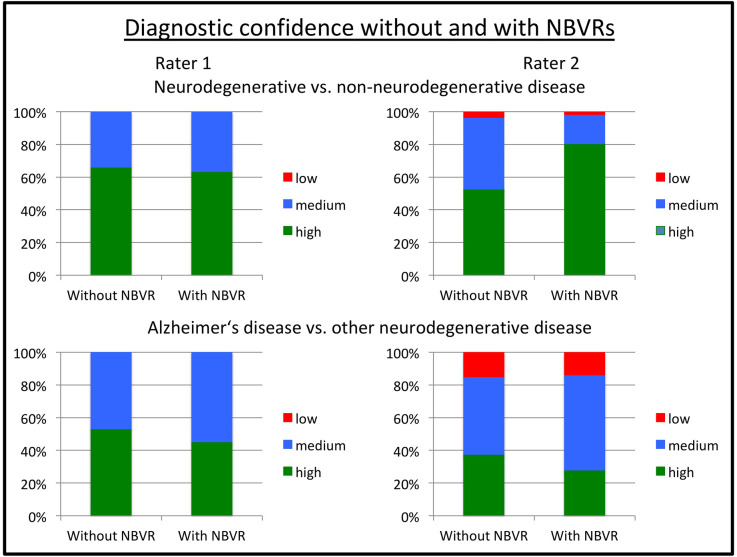
Diagnostic confidence without and with NBVR information. Ratings on a three-point Likert scale ranging from 1 (“high confidence”) to 3 (“low confidence”) are shown for rater 1 (left column) and rater 2 (right column). Diagnostic questions were the identification of any neurodegenerative pattern (upper row) and the identification of a pattern suggestive of Alzheimer’s disease (AD; lower row). We observed increased diagnostic confidence in distinguishing patients with neurodegenerative disorders from patients without evidence of neurodegeneration on FDG-PET for rater 2 (*p* < 0.001). Abbreviations: NBVR, Normative brain volume report; FDG-PET, Fluorodeoxyglucose-Positron Emission Tomography.

In order to measure the individual diagnostic confidence with or without the use of NBVRs, raters assigned a score ranging from 1 (“high confidence”) to 3 (“low confidence”) to each rating. Diagnostic confidence without and with the use of an NBVR for detecting any neurodegenerative disorder was 1.34 (±0.48)/1.37 (±0.48) and 1.51 (±0.57)/1.21 (±0.45) for raters 1 and 2, respectively. Diagnostic confidence without and with the use of an NBVR for detecting an atrophy pattern suggestive of AD was 1.47 (±0.50)/1.55 (±0.50) and 1.77 (±0.70)/1.86 (±0.64) for raters 1 and 2, respectively. Thus, a significant increase in diagnostic confidence for identifying the presence of any neurodegenerative disorder was shown for rater 2 (*p* < 0.001). For visualization, please see Figure 3.

### Interrater reliability

Cohen’s κ was calculated for interrater agreement with respect to: (1) detection of any neurodegenerative disease pattern and (2) detection of AD. Interrater agreement for detection of any neurodegenerative disorder by visual inspection only was poor [Cohen’s *κ* = 0.298 (95%-CI: 0.132–0.446)] and substantially increased for visual inspection plus NBVR [Cohen’s *κ* = 0.560 (95%-CI: 0.394–0.707)]. Interrater agreement for detection of AD by visual inspection only was good [Cohen’s *κ* = 0.557 (95%-CI: 0.187–0.884)] and decreased for visual inspection plus NBVR [Cohen’s *κ* = 0.2907 (95%-CI: −0.025–0.614)].

## Discussion

In this study, we investigated the impact of CE-certified NBVRs with initial clinical validation on the diagnosis of dementia disorders in real-world clinical practice. We observed no significant changes in diagnostic accuracy for both raters. However, one of two raters showed decreased sensitivity at increased specificity for the identification of patients with neurodegenerative disorders. Furthermore, a significant increase in diagnostic confidence was found for one rater, when differentiating between neurodegenerative and non-neurodegenerative disorders. Especially the increased specificity could be important in clinical practice since it improves the validity of the neuroradiology report in case of a positive finding.

The impact of NBVRs on diagnostic accuracy in patients with degenerative disorders has been investigated before. As expected, diagnostic accuracy as well as the impact of NBVRs under real-world clinical conditions were worse than in our previous study in a pre-selected cohort of patients focusing on the differential diagnosis (Hedderich et al., 2020). Whereas the study design with two reading sessions was rather similar, different raters and the different patient populations may in part also cause differences in study outcomes. Very few studies on diagnostic accuracy using CE-marked NBVR tools exist as outlined in a recent review (Pemberton et al., 2021b) and most studies report either the diagnostic accuracy of one or more preselected volumetric measurements (e.g., hippocampus volume) alone or their correlation with corresponding visual assessment (mesial temporal atrophy score; Min et al., 2017; Persson et al., 2017; Koikkalainen et al., 2019). Two studies using CE-marked tools in relatively large cohorts of patients with neurodegenerative disorders found statistically significant separation of AD patients from non-AD dementia patients by automatically derived hippocampus volume (Persson et al., 2017) and moderate accuracy of automated identification of patients with neurodegenerative dementia disorders using support vector machines (Morin et al., 2020). Studying normative brain development and aging has benefited from huge aggregated and harmonized datasets in the last few years. Very recently, Bethlehem et al. (2022) have derived growth charts of the human brain from more than 100,000 participants from *in utero* to 100 years of age and provided a website where new samples can be compared with this benchmark. In addition, other brain volumetry solutions without CE marking have been studied for similar tasks for more than a decade with very promising initial results on selected patient cohorts but more sobering results in a prospective memory clinic setting (Klöppel et al., 2008, 2015). In the most recent and first multi-rater clinical evaluation, quantitative MRI atrophy reports were identified as a potential diagnostic aid for the assessment of patients with neurodegenerative disorders, but, with mixed results (Pemberton et al., 2021a). Including several raters of different experience levels (registrars, consultants, non-clinical image analysts), the authors found increased overall sensitivity and diagnostic accuracy using quantitative reports (Goodkin et al., 2019). Interestingly, on a group-level analysis, the improvement was only statistically significant for consultants (Pemberton et al., 2021a). Overall, these results underline the need for further diagnostic accuracy studies in consecutive, ideally prospective patient cohorts. Our results demonstrate the complexity of integrating NBVRs as an additional piece of information into the clinical decision-making process, possibly with the need for special clinical radiology training. This complexity is further reflected in the evaluation of diagnostic confidence. Rater 2 stated an increase in diagnostic confidence using NBVRs with significantly less “low confidence” diagnostic decisions. However, the diagnostic performance of correct classifications did not improve for rater 2, which shows the complicated relationship between subjective patient classification and diagnostic confidence.

In the present study of local atrophy measurements, we chose ratings of FDG-PET images as the reference standard, thus comparing the interpretation of brain atrophy to brain hypometabolism. It has been shown, that a pattern of hypometabolism on FDG-PET usually precedes brain atrophy in the evolution of neurodegenerative diseases (Grothe and Teipel, 2016). FDG-PET is an established tool to assist in the diagnosis of dementia disorders, e.g., for the early identification of AD typical patterns in patients with mild cognitive impairment (Arbizu et al., 2018) or for the differential diagnosis of distinct neurodegenerative disorders (Nestor et al., 2018), which is reflected in current diagnostic guidelines (Nobili et al., 2018). Interestingly, stereotactic projections of z-score deviations have become much more common in nuclear medicine and are widely used in clinical practice (Minoshima et al., 1995; Minoshima, 2003; Matsunari et al., 2007), whereas similar approaches of MRI postprocessing are not commonly used as of today (Caspers et al., 2021). We found limited value of NBVRs on diagnostic accuracy in the current study sample using FDG-PET as the reference standard. The reasons for this can be considered manifold and may certainly to some extent be caused by true biological differences between hypometabolism and structural atrophy (Grothe and Teipel, 2016). Other reasons may be inherent limitations of our study design pertaining either to technical factors for NBVR generation or to the integration of NBVR information into the neuroradiological-decision making. While it is an advantage for clinical transferability that we have deployed a CE-marked NBVR tool, this also comes with the disadvantage of predefined settings, e.g., with respect to the statistical thresholds to define “abnormal” brain structure, the number of healthy control samples in the normative cohort and the visualization method. Moreover, these results may not be transferable to other CE-marked brain volumetry solutions on the market. All of these factors may be important for the correct delineation of pathologic brain atrophy and should be investigated in future studies. In addition, the dependency of algorithmic output on technical factors should be acknowledged by regulatory authorities, which should give end-users the possibility to adapt technical algorithmic features to the local clinical setting. Choosing FDG-PET as the reference standard, which was available by design in the entire study cohort since we performed PET-MRI, allowed us to include an almost complete consecutive study cohort. While this is desirable for our study design and closer to clinical reality, it also represents a limitation since we were not able to investigate how NBVRs impact the diagnostic accuracy with respect to a gold standard clinical diagnosis (based on imaging studies, biomarker information, clinical evaluation, neurocognitive evaluation, and follow-up visits). However, it was the specific aim of the study to investigate whether MRI-based NBVRs improve the prediction of FDG-PET patterns in our cohort.

It is another limitation of the current study that we have not taken into account how the neuroradiologist deals with the information provided by the NBVR and whether he or she takes it into account for the final decision. The need for a better understanding of the interaction between NBVR and neuroradiologists is underlined by the differential effect of NBVRs on interrater reliability. We saw an increase in Cohen’s kappa with NBVRs for the identification of any neurodegenerative disorder pattern but a strong decrease with NBVRs for the identification of AD-typical patterns of neurodegeneration. But also other questions seem pertinent: At what point does a patch of local atrophy in the stereotactic surface projection seem convincing? Is it size or location? Probably a mixture of both, together with the overall visual impression of additional brain MRI sequences. Additionally, it seems of interest how this differs for neuroradiologists with distinct levels of expertise. Future studies should focus more on these “soft” aspects, pertaining to explainable AI and the interaction between algorithmic information and human decision-making.

In conclusion, this diagnostic accuracy study using a state-of-the-art, CE-marked tool for NBVRs showed a partial impact on diagnostic decision-making and elevated diagnostic confidence in one of two readers. The impact on specificity when diagnosing patients with any neurodegenerative disorder is noteworthy since it was significantly elevated to a subspecialist level, thus improving the validity of the neuroradiology report in case of a positive finding (Pemberton et al., 2021a). We propose that future studies should focus on technical advances to narrow the gap between metabolic and structural imaging and on the dedicated investigation of how NBVR information impacts human decision-making in clinical neuroradiology.

## Data Availability Statement

The data analyzed in this study is subject to the following licenses/restrictions: Brain MRI must not be shared with external partners due to restrictions of data privacy. Requests to access these datasets should be directed to dennis.hedderich@tum.de.

## Ethics Statement

The studies involving human participants were reviewed and approved by Ethics committee of the University Hospital rechts der Isar. Written informed consent for participation was not required for this study in accordance with the national legislation and the institutional requirements. Study procedures were approved by the ethics committee of the Technical University of Munich (622/20 S). Written informed consent was waived due to the retrospective nature of this analysis.

## Author Contributions

DH, IY, BS-K, MS, TG, SS, DS, and VS designed the experiment. DH, BS-K, MS, and IY carried it out. DH, DS, CR, JC, IY, SS, and BS-K analyzed the data. DH, BS-K, MS, and IY wrote the manuscript. DS, VS, CR, JC, TG, CZ, and SS edited the manuscript. CZ, TG, and IY supervised the work. All authors contributed to the article and approved the submitted version.

## References

[B1] Arbizu J. Festari C. Altomare D. Walker Z. Bouwman F. Rivolta J.. (2018). Clinical utility of FDG-PET for the clinical diagnosis in MCI. Eur. J. Nucl. Med. Mol. Imaging 45, 1497–1508. 10.1007/s00259-018-4039-7 29704037

[B2] Ashburner J. (2007). A fast diffeomorphic image registration algorithm. Neuroimage 38, 95–113. 10.1016/j.neuroimage.2007.07.007 17761438

[B3] Ashburner J. Friston K. J. (2000). Voxel-based morphometry—the methods. Neuroimage 11, 805–821. 10.1006/nimg.2000.0582 10860804

[B4] Bethlehem R. A. I. Seidlitz J. White S. R. Vogel J. W. Anderson K. M. Adamson C.. (2022). Brain charts for the human lifespan. Nature 604, 525–533. 10.1038/s41586-022-04554-y 35388223 PMC9021021

[B5] Brown R. K. J. Bohnen N. I. Wong K. K. Minoshima S. Frey K. A. (2014). Brain PET in suspected dementia: patterns of altered FDG metabolism. Radiographics 34, 684–701. 10.1148/rg.343135065 24819789

[B6] Bruun M. Frederiksen K. S. Rhodius-Meester H. F. M. Baroni M. Gjerum L. Koikkalainen J.. (2019). Impact of a clinical decision support tool on prediction of progression in early-stage dementia: a prospective validation study. Alzheimers. Res. Ther. 16, 91–101. 10.1186/s13195-019-0482-3 30894218 PMC6425602

[B7] Caspers J. Heeger A. Turowski B. Rubbert C. (2021). Automated age- and sex-specific volumetric estimation of regional brain atrophy: workflow and feasibility. Eur. Radiol. 31, 1043–1048. 10.1007/s00330-020-07196-8 32852588 PMC7813701

[B8] Forman S. D. Cohen J. D. Fitzgerald M. Eddy W. F. Mintun M. A. Noll D. C. (1995). Improved assessment of significant activation in functional magnetic resonance imaging (fMRI): use of a cluster-size threshold. Magn. Reson. Med. 33, 636–647. 10.1002/mrm.1910330508 7596267

[B9] Frisoni G. B. Fox N. C. Jack C. R. J. Scheltens P. Thompson P. M. (2010). The clinical use of structural MRI in Alzheimer disease. Nat. Rev. Neurol. 6, 67–77. 10.1038/nrneurol.2009.215 20139996 PMC2938772

[B10] Goodkin O. Pemberton H. Vos S. B. Prados F. Sudre C. H. Moggridge J.. (2019). The quantitative neuroradiology initiative framework: application to dementia. Br. J. Radiol. 92:20190365. 10.1259/bjr.20190365 31368776 PMC6732931

[B11] Grothe M. J. Teipel S. J. (2016). Spatial patterns of atrophy, hypometabolism and amyloid deposition in Alzheimer’s disease correspond to dissociable functional brain networks. Hum. Brain Mapp. 37, 35–53. 10.1002/hbm.23018 26441321 PMC4715545

[B12] Hedderich D. M. Dieckmeyer M. Andrisan T. Ortner M. Grundl L. Schön S.. (2020). Normative brain volume reports may improve differential diagnosis of dementing neurodegenerative diseases in clinical practice. Eur. Radiol. 30, 2821–2829. 10.1007/s00330-019-06602-0 32002640

[B13] Huppertz H.-J. Kroll-Seger J. Kloppel S. Ganz R. E. Kassubek J. (2010). Intra- and interscanner variability of automated voxel-based volumetry based on a 3D probabilistic atlas of human cerebral structures. Neuroimage 49, 2216–2224. 10.1016/j.neuroimage.2009.10.066 19878722

[B14] Klöppel S. Peter J. Ludl A. Pilatus A. Maier S. Mader I.. (2015). Applying automated MR-based diagnostic methods to the memory clinic: a prospective study. J. Alzheimers. Dis. 47, 939–954. 10.3233/JAD-150334 26401773 PMC4923764

[B15] Klöppel S. Stonnington C. M. Barnes J. Chen F. Chu C. Good C. D.. (2008). Accuracy of dementia diagnosis—a direct comparison between radiologists and a computerized method. Brain 131, 2969–2974. 10.1093/brain/awn239 18835868 PMC2577804

[B16] Koikkalainen J. R. Rhodius-Meester H. F. M. Frederiksen K. S. Bruun M. Hasselbalch S. G. Baroni M.. (2019). Automatically computed rating scales from MRI for patients with cognitive disorders. Eur. Radiol. 29, 4937–4947. 10.1007/s00330-019-06067-1 30796570

[B17] Malone I. B. Leung K. K. Clegg S. Barnes J. Whitwell J. L. Ashburner J.. (2015). Accurate automatic estimation of total intracranial volume: a nuisance variable with less nuisance. Neuroimage 104, 366–372. 10.1016/j.neuroimage.2014.09.034 25255942 PMC4265726

[B18] Matsunari I. Samuraki M. Chen W.-P. Yanase D. Takeda N. Ono K.. (2007). Comparison of ^18^F-FDG PET and optimized voxel-based morphometry for detection of Alzheimer’s disease: aging effect on diagnostic performance. J. Nucl. Med. 48, 1961–1970. 10.2967/jnumed.107.042820 18006622

[B19] Min J. Moon W.-J. Jeon J. Y. Choi J. W. Moon Y.-S. Han S.-H. (2017). Diagnostic efficacy of structural MRI in patients with mild-to-moderate Alzheimer disease: automated volumetric assessment versus visual assessment. Am. J. Roentgenol. 208, 617–623. 10.2214/AJR.16.16894 28075620

[B20] Minoshima S. (2003). Imaging Alzheimer’s disease: clinical applications. Neuroimaging Clin. N. Am. 13, 769–780. 10.1016/s1052-5149(03)00099-6 15024960

[B21] Minoshima S. Frey K. A. Koeppe R. A. Foster N. L. Kuhl D. E. (1995). A diagnostic approach in Alzheimer’s disease using three-dimensional stereotactic surface projections of fluorine-18-FDG PET. J. Nucl. Med. 36, 1238–1248. 7790950

[B22] Morin A. Samper-Gonzalez J. Bertrand A. Ströer S. Dormont D. Mendes A.. (2020). Accuracy of MRI classification algorithms in a tertiary memory center clinical routine cohort. J. Alzheimer’s Dis. 74, 1157–1166. 10.3233/JAD-190594 32144978

[B23] Mühlau M. Wohlschlager A. M. Gaser C. Valet M. Weindl A. Nunnemann S.. (2009). Voxel-based morphometry in individual patients: a pilot study in early Huntington disease. Am. J. Neuroradiol. 30, 539–543. 10.3174/ajnr.A1390 19074546 PMC7051432

[B24] Nestor P. J. Altomare D. Festari C. Drzezga A. Rivolta J. Walker Z.. (2018). Clinical utility of FDG-PET for the differential diagnosis among the main forms of dementia. Eur. J. Nucl. Med. Mol. Imaging 45, 1509–1525. 10.1007/s00259-018-4035-y 29736698

[B25] Nobili F. Arbizu J. Bouwman F. Drzezga A. Agosta F. Nestor P.. (2018). European association of nuclear medicine and european academy of neurology recommendations for the use of brain ^18^F-fluorodeoxyglucose positron emission tomography in neurodegenerative cognitive impairment and dementia: delphi consensus. Eur. J. Neurol. 25, 1201–1217. 10.1111/ene.13728 29932266

[B26] Opfer R. Suppa P. Kepp T. Spies L. Schippling S. Huppertz H. J. (2016). Atlas based brain volumetry: how to distinguish regional volume changes due to biological or physiological effects from inherent noise of the methodology. Magn. Reson. Imaging 34, 455–461. 10.1016/j.mri.2015.12.031 26723849

[B27] Pemberton H. G. Goodkin O. Prados F. Das R. K. Vos S. B. Moggridge J.. (2021a). Automated quantitative MRI volumetry reports support diagnostic interpretation in dementia: a multi-rater, clinical accuracy study. Eur. Radiol. 31, 5312–5323. 10.1007/s00330-020-07455-8 33452627 PMC8213665

[B28] Pemberton H. G. Zaki L. A. M. Goodkin O. Das R. K. Steketee R. M. E. Barkhof F.. (2021b). Technical and clinical validation of commercial automated volumetric MRI tools for dementia diagnosis-a systematic review. Neuroradiology 63, 1773–1789. 10.1007/s00234-021-02746-3 34476511 PMC8528755

[B29] Persson K. Selbæk G. Brækhus A. Beyer M. Barca M. Engedal K. (2017). Fully automated structural MRI of the brain in clinical dementia workup. Acta Radiol. 58, 740–747. 10.1177/0284185116669874 27687251

[B30] Potvin O. Dieumegarde L. Duchesne S. (2017). Normative morphometric data for cerebral cortical areas over the lifetime of the adult human brain. Neuroimage 156, 315–339. 10.1016/j.neuroimage.2017.05.019 28512057

[B31] Rutherford S. Fraza C. Dinga R. Kia S. M. Wolfers T. Zabihi M.. (2022). Charting brain growth and aging at high spatial precision. eLife 11:e72904. 10.7554/eLife.72904 35101172 PMC8828052

[B32] Teipel S. Drzezga A. Grothe M. J. Barthel H. Chételat G. Schuff N.. (2015). Multimodal imaging in Alzheimer’s disease: validity and usefulness for early detection. Lancet Neurol. 14, 1037–1053. 10.1016/S1474-4422(15)00093-9 26318837

[B33] Teipel S. Kilimann I. Thyrian J. R. Kloppel S. Hoffmann W. (2017). Potential role of neuroimaging markers for early diagnosis of dementia in primary care. Curr. Alzheimer Res. 15, 18–27. 10.2174/1567205014666170908093846 28891447

[B34] Vernooij M. W. Pizzini F. B. Schmidt R. Smits M. Yousry T. A. Bargallo N.. (2019). Dementia imaging in clinical practice: a European-wide survey of 193 centres and conclusions by the ESNR working group. Neuroradiology 61, 633–642. 10.1007/s00234-019-02188-y 30852630 PMC6511357

[B35] Whitwell J. L. Jack C. R. J. Przybelski S. A. Parisi J. E. Senjem M. L. Boeve B. F.. (2011). Temporoparietal atrophy: a marker of AD pathology independent of clinical diagnosis. Neurobiol. Aging 32, 1531–1541. 10.1016/j.neurobiolaging.2009.10.012 19914744 PMC2888989

[B36] Yakushev I. Ripp I. Wang M. Savio A. Schutte M. Lizarraga A.. (2022). Mapping covariance in brain FDG uptake to structural connectivity. Eur. J. Nucl. Med. Mol. Imaging 49, 1288–1297. 10.1007/s00259-021-05590-y 34677627 PMC8921091

